# ‘Placement budgets’ for supported employment – improving competitive employment for people with mental illness: study protocol of a multicentre randomized controlled trial

**DOI:** 10.1186/1471-244X-12-165

**Published:** 2012-10-04

**Authors:** Carlos Nordt, Elisabeth Brantschen, Wolfram Kawohl, Bettina Bärtsch, Helene Haker, Nicolas Rüsch, Wulf Rössler

**Affiliations:** 1 Psychiatric University Hospital Zurich, Research Unit for Clinical and Social Psychiatry, Lenggstrasse 31, PO Box 1931, 8032, Zürich, Switzerland

**Keywords:** Mental illness, Supported employment, Randomized controlled trial, Social network, Social support, Stigmatization, Switzerland

## Abstract

**Background:**

Vocational integration of people with mental illness is poor despite their willingness to work. The ‘Individual Placement and Support’ (IPS) model which emphasises rapid and direct job placement and continuing support to patient and employer has proven to be the most effective vocational intervention programme. Various studies have shown that every second patient with severe mental illness was able to find competitive employment within 18 months. However, the goal of taking up employment within two months was rarely achieved. Thus, we aim to test whether the new concept of limited placement budgets increases the effectiveness of IPS.

**Methods/Design:**

Six job coaches in six out-patients psychiatric clinics in the Canton of Zurich support unemployed patients of their clinic who seek competitive employment. Between June 2010 and May 2011 patients (N=100) are randomly assigned to three different placement budgets of 25h, 40h, or 55h working hours of job coaches. Support lasts two years for those who find a job. The intervention ends for those who fail to find competitive employment when the respective placement budgets run out. The primary outcome measure is the time between study inclusion and first competitive employment that lasted three months or longer. Over a period of three years interviews are carried out every six months to measure changes in motivation, stigmatization, social network and social support, quality of life, job satisfaction, financial situation, and health conditions. Cognitive and social-cognitive tests are conducted at baseline to control for confounding variables.

**Discussion:**

This study will show whether the effectiveness of IPS can be increased by the new concept of limited placement budgets. It will also be examined whether competitive employment leads in the long term to an improvement of mental illness, to a transfer of the psychiatric support system to private and vocational networks, to an increase in financial independence, to a reduction of perceived and internalized stigma, and to an increase in quality of life and job satisfaction of the patient. In addition, factors connected with fast competitive employment and holding that job down in the long term are being examined (motivation, stigmatization, social and financial situation).

**Trial register:**

ISRCTN89670872

## Background

Unemployment rates are high among people with mental illness despite available vocational rehabilitation and their willingness to work [1]. As an alternative to traditional vocational rehabilitation (train-and-place), supported employment emphasises rapid and direct job placements and support of patients and employer (place-and-train) [2]. A higher effectiveness of ‘Individual Placement and Support’ (IPS), an intensively studied supported employment intervention in the USA, than of vocational rehabilitation has also been proven in a randomized controlled trial in six European countries with differing labour markets and welfare systems [3,4].

In the IPS model, a job coach is an integral member of a mental health care team who helps patients to find competitive employment corresponding to their wishes as soon as possible, and who continues to support them and their employer, so that jobs can be held down as long as possible. Various studies have shown that for every second patient with severe mental illness competitive employment could be found within 18 months [2]. However, the goal of taking up employment within two 2 months was rarely achieved. The main question of this study, therefore, is whether limiting the ‘Placement-Budget’ leads to a faster take-up of competitive employment, thereby resulting in a better utilization of the overall resources of a job coach.

### Placement budgets

The idea of placement budgets is new, probably because IPS studies addressed the group of people with serious mental illness, i.e. those who hardly seem to have a chance for competitive employment. Such high moral standing seems incompatible with discussions on limits of cost or of time needed for IPS interventions. However, as IPS has proven to be effective for people with serious mental illness, why should unemployed patients with milder forms of mental illness be excluded from IPS interventions? If, for example, all patients of an out-patient facility of a psychiatric hospital can receive IPS interventions, it is inevitable that the resources invested must be justified towards cost providers. Moreover, seen from the perspective of a cost provider, it is not very attractive to fund an intervention in which every patient will need a job coach for 18 months, but with only a 50 percent chance of finding competitive employment for one day or longer. If competitive employment is found, it is probably much easier to convince cost providers that a budget for support is a meaningful investment, so that the job coach can assist with long-term vocational integration.

According to the IPS model, the caseload of a full-time job coach should not exceed 25 clients [2]. Thus, using resources for clients who did not find a competitive employment after a longer time period reduces the resources available to the job coach for the placement of new clients and helping other clients to keep their competitive employment.

### Meaningful duration of the intervention

The new concept of placement budget can be understood as the question of how much of an intervention is initially needed. The next question from the standpoint of a cost provider doubtlessly concerns the meaningful duration of an intervention. Therefore, in order to ascertain whether a period of up to two years of support by a job coach is sufficient to stabilize the vocational integration and ensure that it will not change during the year after the end of the intervention, we set the time frame of the study accordingly. A period of up to two years of support by a job coach is longer than the duration of support in most trials, which typically last 18 months [3]. Moreover, those trials usually have no follow-up after the intervention period, probably due to the last core principle of supported employment enumerated by Crowther et al. [3]: ‘(*f*) follow on support is continued indefinitely’.

### Outcomes

Other research questions focus on clients’ outcomes. For most individuals there are more advantages to competitive employment than disadvantages and there is evidence that the concerns among clinicians about possible detrimental effects of working and supported employment are out of place [1]. Working is important in several ways. Unemployment deprives people of the social and psychological functions of work such as social support and structuring of time [5,6]. Being without work and a regular income puts people at risk of poverty, even if they receive social benefits [7]. But vocational integration does not always mean financial independence, as some clients of supported employment have unskilled jobs and work only a few hours per week [2]. There might be even a benefit trap, e.g. a loss of disability payments upon returning to work [1,2].

### Predictors for rapid job placement

Overall, it is not well understood which clients’ chances of finding competitive employment are higher. A meta analysis found only very small effect sizes for age, gender, race, and diagnosis [8]. Even having prior employment, one of the strongest predictors in many studies, did not result in a significant effect in every study [8]. The study in the six European countries tested a variety of socio-demographic and illness-related variables as predictors for entering competitive employment but found a substantial effect only for previous work history [9].

### Motivation

In opposition to socio-demographic and illness-related factors there is a lack of research with respect to the client motivation. This is surprising as it has been repeatedly stated that the motivation of the client is central to the success of IPS [2]. One reason for the lack of research on factors influencing client motivation could be that ‘much of the body of psychiatric rehabilitation research consists of atheoretical empirical investigations’ [8]. The theory of reasoned action of Fishbein and Ajzen [10], one of the most important theories on the relationship between attitude and behaviour, is suitable to be applied to supported employment. Not only the attitude of the client, i.e. the self-rated importance of obtaining competitive employment, but also the subjective norms of the social environment probably influence the intention to find and to maintain competitive employment. Here subjective norms means how the client thinks that significant others view client’s being competitively employed, weighted by the importance of those attitudes to the client.

### Stigma

According to the Modified Labeling Theory of Link et al. [11] people with mental illness avoid potentially stigmatizing situations if they believe that people with mental illness are generally discriminated. Thus, people who score high on the perceived discrimination scale could probably have a low vocational outcome. Moreover, we observed for people with a more recent onset of illness that how they had perceived the social support of their social network during their inpatient treatment modified their perceived discrimination score [12]. Thus, new experiences in a competitive employment situation could probably also modify perceived discrimination.

Over the last decade there has been a growing interest in the subjective experience of stigma among people with mental illness and on the relationship of stigma and work [13]. We therefore are interested whether IPS has an influence on the appraisal of stigma-related stress [14], and on the internalization of the stigma of mental illness [15].

### Social-cognitive tests for interaction skills

Among job coaches there is often considerable variance in employment outcomes within supported employment programmes [16]. These differences appear to be related to their clinical skills. Those skills comprise specific interactions (transactions with clients, with other staff members, and with employers) and their activity during stages of supported employment (engagement, assessment, finding a job that matches talents and interests, insuring success by addressing skills and supports, leaving a job appropriately, and finding another job) [16]. As in most trials the number of job coaches is very small [3], it is almost impossible to find general factors that could explain the variance among them with regard to employment outcomes. As we aim to explore the influence of social-cognitive abilities of clients on employment outcomes, the six job coaches also performed those social-cognitive tests, as this might partly explain their clinical skills. These tests consist of a prosody test (affect recognition in speech according to the methodological propositions of Edwards and colleagues [17]), the reading-the-mind-in-the-eyes test [18] to measure the ability to visually recognize complex emotions in pictures, an attribution style test [19], and a socio-physiological test of resonance capability in terms of contagion by yawning or laughing [20]. Four separate aspects of empathy are assessed using the Interpersonal Reactivity Index [21].

### Research questions

The main objective of this study is to assess whether the effectiveness of IPS can be improved by using limited placement budgets. If this is true, the job coach could invest more working hours to support both the patient on the job and his/her employer. Thus, the main study hypotheses are:

Primary: The more limited the amount of working hours available to a job coach to find a job, the faster a placement in open, competitive employment.

Secondary: Ascertain factors for fast job placement and long-term job tenure. Primary issues are motivation, stigmatization, social network and social support, quality of life, job satisfaction, financial situation, and health conditions. Cognitive and social-cognitive tests will be conducted to control for confounding variables.

## Methods

This is a multicentre randomized controlled trial in the following six out-patient psychiatric clinics in the Canton of Zurich: Ambulatorium Oerlikon (PUK Zürich), Zentrum für Gemeinde- und Familienpsychiatrie (ZGF, PUK Zürich), Psychiatrisches Ambulatorium Zimmerberg (Sanatorium Kilchberg), Psychiatrische Poliklinik Winterthur (ipw), Kriseninterventionszentrum (KIZ, PUK Zürich), Gemeindepsychiatrisches Zentrum Bülach (ipw). Job coaches work part-time (50%) as integral members of the mental health care team and recruit participants from the caseloads of their out-patient centre. Patients who are interested in the study are provided with verbal and written information about the subject-matter of the study and the criteria for participation. After they have given informed consent the participants (N=100) are randomly assigned to one of the three different placement budgets (25h, 40h, and 55h working hours of job coach). These placement budgets were thought to be rather small (25h), adequate (40h), or rather large (55h) by the supervisor (BB) of our supported employment department. To minimize effects due to different employment outcomes of the job coaches, block randomization with a block size of six was chosen, so that for each job coach, all three budget groups will be similarly sized.

Placement budget allocation could not be blinded to participants, job coaches, and researchers. On the contrary, job coaches and participants had to discuss how best to invest the allocated placement budget. Job coaches will support the clients for up to two years, or until the placement budget has run out for those who failed to find competitive employment. Irrespective of whether participants are still supported by the job coach, assessments are carried out every six months over a period of three years (t0-t6).

Job coaches are trained in the IPS model and will have weekly meetings with supervision at the Supported Employment Department of the Psychiatric University Hospital established in 2003. A web based software programme was tailored with a schedule tool for job coaches that automatically computes their remaining placement budgets. In the schedule tool the job coaches have to add one of the following labels to each entry: ‘initial meeting’ (to inform about the study, to assist with inclusion criteria, to obtain informed consent), ‘placement budget’ (time needed for engagement, assessment, and to find a job that matches the talents and interests of a specific client), ‘support budget’ (time needed to support competitive employment of a specific client), ‘job acquisition’ (time needed to find or develop competitive employment without a specific client in mind), ‘team’ (time needed as a member of the mental health care team), ‘supervision’ (time needed to attend the supported employment meetings), ‘other or unspecified’ for the remaining time. The web-based software also included all instruments, as well as a timing function to remind the interviewers two weeks before the next scheduled assessment.

Before the start of recruitment in June 2010, the study protocol was approved by Zurich Cantonal Ethics Committee (CEC), Division 3 (Kantonale Ethik-Kommission Zürich (KEK) Abteilung 3), reference number E-51/2009.

### Inclusion and exclusion criteria

Screening for inclusion and exclusion criteria is carried out by the job coaches.

*Inclusion criteria*

1.Current treatment in one of the six participating out-patient psychiatric clinics

2.Twelve months of unemployment and no programme of vocational integration over the last three months

3.Motivation to work in competitive employment

4.Being of working age (18–60 years)

5.Resident in the Canton of Zurich

6.Willing and capable of giving informed consent

*Exclusion criteria*

1.Severe organic illness (ICD-10, F0)

2.Insufficient knowledge of German.

### Instruments

To enhance long-term participation we used a limited number of questionnaires in the face-to-face interviews and selected instruments that were well accepted in previous studies. Not all instruments are used both at the baseline (t0) as well as at the six follow-up (t1-t6) interviews.

#### Self-rated by the participant (in chronological order)

• Motivation (t0-t6 interviews), newly developed questionnaire following the theory of reasoned action of Fishbein and Ajzen [10]: Self-rated importance of obtaining competitive employment; how the client thinks that significant others view the client’s being competitively employed and how important these attitudes are to the client. All items are rated on a five-point scale ranging from 1 ‘very unimportant’ to 5 ‘very important’. Significant others were: spouse/partner, relatives with whom they have a close relationship, close friends, children, mental health worker with whom they have a close relationship.

• Living situation (t0-t6 interviews): part of the Client Socio-demographic and Service Receipt Inventory, CSSRI-EU [22], German translation

• Social network diversity and support (t0-t6 interviews): LUNST scales [23]; with additional questions on instrumental and emotional support from a mental health worker with whom they have a close relationship

• Perceived discrimination (t0-t6 interviews) [11]

• Discrimination experiences on the job over the last 12 months (t2 interview): five self-developed items to be answered on a seven-point scale ranging from 1 ‘completely disagree’ to 7 ‘completely agree’.

• Education, profession, vocational experience (t0 interview): part of the Client Socio-demographic and Service Receipt Inventory, CSSRI-EU [22], German translation

• Employment status, duration, job description - separate for competitive or sheltered employment over the last six months (t1-t6 interviews)

• Job Satisfaction (t1-t6 interviews): Indiana Job Satisfaction Scale, IJSS [24], German translation

• Income sources (t0-t6 interviews): part of the Client Socio-demographic and Service Receipt Inventory, CSSRI-EU [22], German translation

• Quality of life (t0-t6 interviews): WHO QoL Bref [25]

• Illness onset, first hospitalization (t0 interview): part of the Client Socio-demographic and Service Receipt Inventory, CSSRI-EU [22], German translation

• Health care utilization of inpatient- and out-patient clinics over the last six months (t0-t6 interviews): part of the Client Socio-demographic and Service Receipt Inventory, CSSRI-EU [22], German translation

• Symptom checklist (t0-t6 interviews): BSI-53 [26]

• Self-esteem (t0, t2 interviews): RSE [27]

• Appraisal of stigma-related stress (t0, t2 interviews): [14],

• Internalization of the stigma of mental illness (t0, t2 interviews): ISMI [15].

#### Cognitive tests at baseline interview (t0)

• Processing speed: Digit Symbol-Coding WAIS-III [28]

• Memory: Digit Span WAIS-III [28]

• Executive functions: Word Fluency Test (Regensburger Wortflüssigkeits-Test) [29]

• Interference suppression: Stroop-Test [30]

• Memory test: Verbal-Learning-and-Memory-Test (Verbaler Lern- und Merkfähigkeitstest) [31]

#### Tests at the centre for neuro- and socio-physiology after baseline interview

• Prosody test: affect recognition in speech according to the methodological propositions of Edwards and colleagues [17]

• Reading-the-mind-in-the-eyes test: ability to recognize complex emotions [18]

• Attribution Style Test [19]

• Resonance Test: contagion by yawning and laughing [20].

• Interpersonal Reactivity Index IRI: four separate aspects of empathy [21]

#### Clinical records at baseline (t0)

As psychiatric hospitals are legally mandated to report admissions to the Central Psychiatric Register (PSYREC) of the Canton of Zurich, we take the following information from the clinical records:

• ICD-10 diagnosis [32]

• Global Assessment of Functioning (GAF) [33]

• Clinical Global Impression (CGI) [34]

#### Ratings by the mental health care staff at baseline (t0)

• Occupational functioning in people with mental health problems: Mini-ICF [35]

• Obvious social stigma (slowdown of movement/motion and language, physical stigma, belonging to a fringe group), open question.

#### Ratings by study coordinator/job coach supervisor (every 3 months)

• IPS Fidelity Scale [2], German translation

#### Information by job coach (as long as participants are in IPS)

• Job status for each instance of competitive employment: job description, exact date of start and end of job and interruption periods, working days per week, total working hours per week, all who helped to find the job (clients themselves, job coach, vocational rehabilitation, recruitment agency, others)

### Data analyses

The primary outcome criterion will be analyzed by means of Cox regression, further outcomes by means of random coefficient models. Both methods allow the examination of multiple predictors and they can appropriately handle dropouts, missing data and different periods between follow-up interviews [36,37]. At a group size of N=33 for each placement budget, the minimal detectable hazard ratio for two-group comparison is 2.3, with a power of 0.8, a significance level of 0.05 (two-sided), an accrual interval of 12 months, a follow-up interval of 24 months and a median time of 12 months.

## Discussion

As unemployment rates are high among people with mental illness despite their willingness to work, we believe that our study will help to reintegrate some of them into society by obtaining competitive employment. The effectiveness of well-implemented IPS programmes is higher than that of other vocational rehabilitation programmes [38]. With the new concept of placement budgets we hope to improve the IPS model in several ways. If the study confirms our hypothesis that a more limited placement budget leads to faster take-up of competitive employment, this would be in the interest of those affected, as well as of the job coaches, the supported employment teams and the mental health care teams. As the financing of supported employment services is still one of the greatest issues [39], the perspective of cost providers is important and placement budgets could be probably an interesting tool for them, these being clearly better than paying for an intervention of unlimited duration but with only a 50% chance of a positive outcome (to have competitive employment for one day or longer within 18 months).

By applying different theories from the social sciences we hope to improve our understanding of which patients have higher chances to find and maintain competitive employment. Some results could lead to new issues that should be addressed by the job coaches or the mental health care team – for example, if we were to find that clients with a strong belief that they are generally discriminated as people with mental illness indeed have a lower vocational outcome. Or if subjective norms of the social network proved to have a great impact on clients’ motivation to work, this could probably be used in a structured way as a resource to improve vocational outcomes.

There are also important limitations to our study. The number of participants in this study is too low to permit conclusions as to which placement budget would be best or adequate for which client group. Moreover, before implementing limited placement budgets there must be consensus regarding their definition and measurement. There must also be evidence that such limitation of placement budgets does not have detrimental effects on clients’ motivation and on the work conditions of the job coaches. Even if we believe that costs and optimal use of resources are important issues, we should not forget that this study is only a first step in a new direction where we directly address limited budgets in a field where the target group are people with serious mental illness whose lives are so difficult that they deserve as much help as they need.

## Abbreviations

IPS: Individual placement and support.

## Competing interests

The authors declare that they have no competing interests.

## Authors’ contributions

CN designed the study, leads the study, performed the statistical analysis and drafted the manuscript. EB coordinate the study, participated in its design and helped to draft the manuscript. WK is co-leader of the study, participated in the design and the organization of the intervention and helped to draft the manuscript. BB is the supervisor of the job coaches and participated in the design of the study. HH designed the social-cognitive and socio-physiological tests. NR designed the tests of the subjective experience of stigma. WR drafted the first study design and is the leader of all six sub-projects of the Zurich Program for Sustainable Development of Mental Health Services (ZInEP). All authors read and approved the final manuscript.

## Pre-publication history

The pre-publication history for this paper can be accessed here:

http://www.biomedcentral.com/1471-244X/12/165/prepub
